# Femoral shaft osteotomy for obligate outward rotation due to SCFE

**DOI:** 10.1007/s11751-017-0276-8

**Published:** 2017-02-22

**Authors:** Peter M. Stevens, Lucas Anderson, Bruce A. MacWilliams

**Affiliations:** Department of Orthopaedics, University of Utah, Salt Lake City, UT 84113 USA

**Keywords:** Slipped capital femoral epiphysis, SCFE, Femoral retroversion, FAI, Femoroacetabular impingement, Femoral osteotomy

## Abstract

Slipped capital femoral epiphysis (SCFE) is an adolescent disease that leads to retroversion of the femoral neck and shaft, relative to the head. Observing that patients with SCFE must walk with an outward foot progression angle and externally rotate the leg in order to flex the hip, we have been performing a femoral shaft rotational osteotomy wherein we rotate the lower femur 45° inward, relative to the upper femur. By correcting retroversion, our goal is to improve functional hip and knee motion, thereby mitigating the effects of SCFE impingement. This is a retrospective review of five hips in four patients (two boys and two girls), average age 14.7 years (range 11 + 7–18 years) who underwent femoral midshaft rotational osteotomy for correction of acquired retroversion of the femur secondary to severe SCFE. We compared clinical findings at the outset to those at an average follow-up of 46 months (range 24–74 months). Pre- and post-gait analysis was performed in three patients. Two of the patients underwent elective arthroscopic osteochondroplasty to alleviate residual FAI: contralateral arthroscopy is pending in one. The first patient in this series received a hip arthroplasty, 62 months after his osteotomy, at age 23. Following midshaft osteotomy, all patients experienced improvement in comfort, gait and activities of daily living. With the patella neutral, they had improved range of hip flexion from an average preoperative flexion of <25° to a postoperative flexion of >90°. Two patients (both male) had delayed union and some loss of correction, secondary to broken interlocking screws; each healed with reamed, exchange nailing. The interlocking screws have since been redesigned and enlarged. Femoral shaft rotational osteotomy restores the functional range of hip motion, while correcting obligate out-toeing and improving knee kinematics. This procedure is technically straightforward, permitting progressive weight bearing, while avoiding the risk of AVN. Osteochondroplasty for residual FAI can be deferred, pending the outcome.

*Level of evidence* III: retrospective series—no controls.

## Introduction

**Fig. 1:**
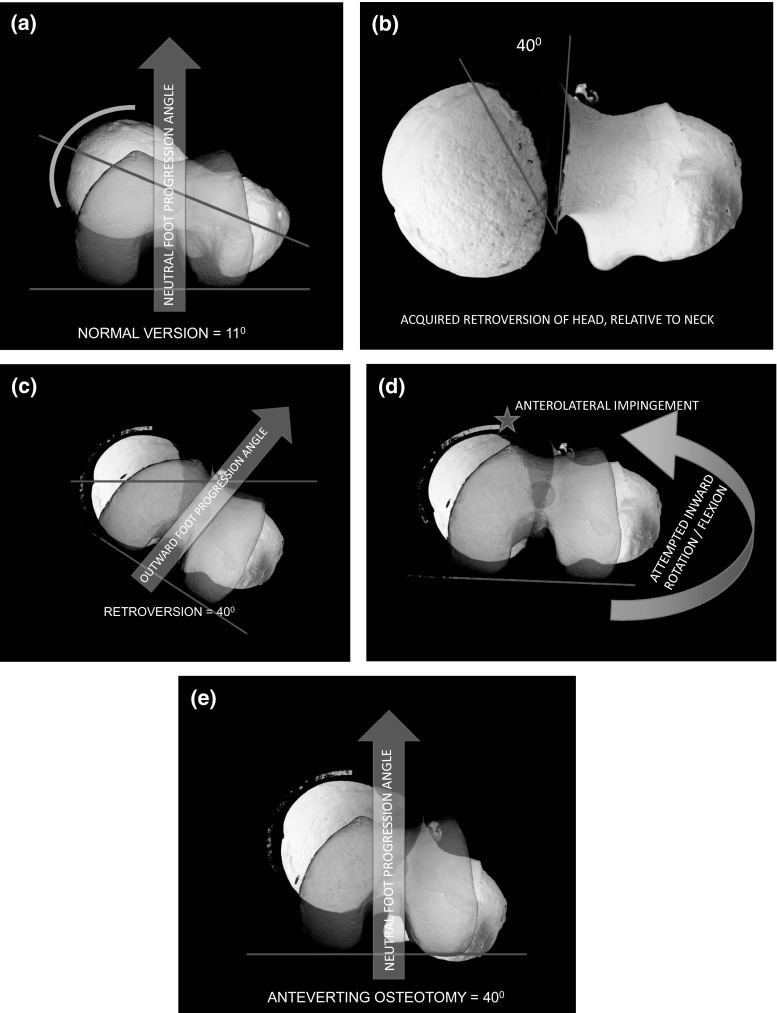
**a** Normal version of the adolescent or adult femur = 11° and the foot progression angle is neutral. **b** Acquired retroversion (40° depicted) may be under-recognized because this is interpreted as varus or extension on plain radiographs. **c** The clinical manifestations include outward foot progression angles noted during gait. **d** Attempted inward rotation of the hip causes anterolateral impingement, exacerbated by attempted hip flexion. This produces an obligatory outward rotation when walking or sitting. **e** The rationale for an anteverting osteotomy is shown here, mitigating the impingement in flexion while restoring the neutral foot progressing angle and improving knee kinematics

Slipped capital femoral epiphysis (SCFE) is an injury to the femoral capital physis. The metaphysis displaces anterosuperiorly relative to the femoral head and produces a varus and retroverted proximal femoral deformity. This deformity can lead to femoroacetabular impingement (FAI) where, in hip flexion, the anterior head–neck junction abuts the anterolateral labrum resulting in pathological compressive and shearing forces to the labral tissues and peripheral cartilage (Fig. 1). The pathomechanics associated with FAI secondary to SCFE has been shown to cause chondrolabral damage in the form of labral degeneration and peripheral acetabular cartilage damage [1–3]. In symptomatic FAI related to SCFE, treatment options range from arthroscopic osteochondroplasty, surgical dislocation and osteochondroplasty (SDO), subcapital realignment, intertrochanteric flexion—valgus osteotomy—as well as hip arthroplasty.

The retroversion associated with severely malunited capital epiphyses not only can result in functional limitations in hip flexion and inward rotation but, additionally, produces an outward foot progression angle during ambulation. This compromises knee kinematics. Patients with bilateral SCFE may have difficulty with daily activities including sitting upright in a chair, tying their shoes, riding a bicycle and driving a car. They complain of knee pain often in addition to hip pain and limited motion.

Consequent to the negative impact of the hip retroversion, we have adopted a strategy for managing symptomatic malunited SCFE by a midshaft and 45^°^ inward rotation femoral shaft osteotomy (FSRO). The goal is to restore a functional range of motion for the involved hip and ipsilateral knee and restore a foot-forward gait. An osteotomy of the proximal femur is avoided and arthroscopic treatment of FAI can be pursued electively. The purpose of this study was to report on the preliminary outcome of a series of SCFE patients treated with an anteverting midshaft osteotomy of the femur for outward rotation in flexion and gait in lieu of the traditional femoral neck or intertrochanteric osteotomy.

## Patients and methods

Between January 2003 and January 2012, four patients (five limbs) with SCFE were treated with femoral shaft rotational osteotomies (FSRO). There were two female and two male patients (one male bilateral FRSO), with an average age of 14.7 years old (range 11 + 7–18 years old) at time of surgery. This retrospective study was approved by the Institutional Board Review.

Three of the hips had prior in situ pinning of their SCFE; the other two had not had any prior intervention for unrecognized SCFE. The indication for surgery in this group of patients included acquired gait disturbance (obligate out-toeing), marked limitation of hip flexion (<25°) and hip or knee pain that interfered with activities of daily living. One patient had mild chondrolysis at presentation (diagnosed from 1 mm of relative narrowing of the articular clear space as compared to the uninvolved hip). This was not exacerbated post-osteotomy.

### Clinical

**Fig. 2:**
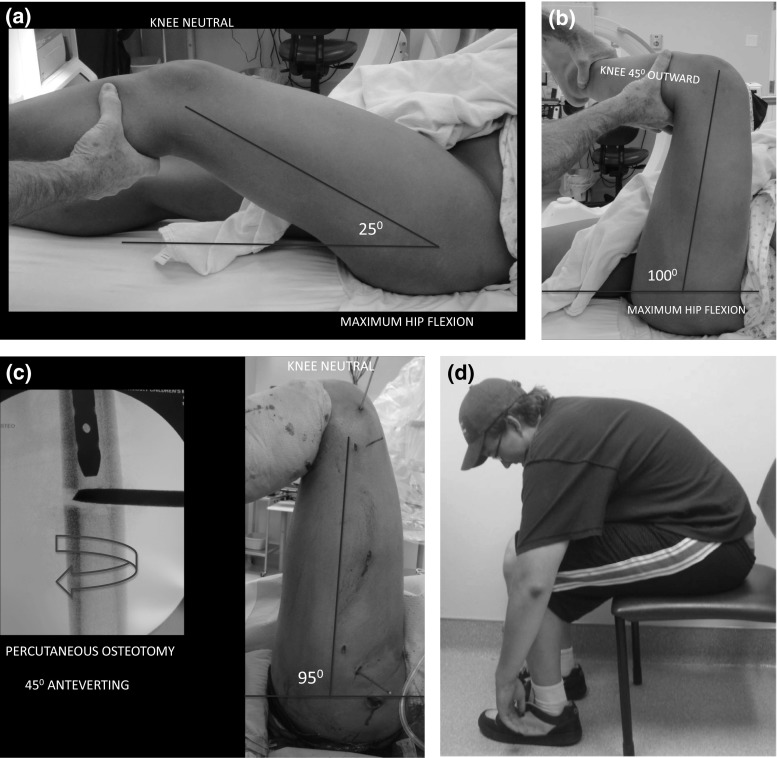
**a** Preoperative attempt to flex the hip with the knee held neutral demonstrates the blockage due to FAI. **b** By simply rotating the hip outward 40°, hip flexion is permitted past 90°. **c** The anteverting femoral osteotomy resolves the clinical problems at the knee while improving functional ROM of the hip. **d** Functional range of hip flexion, maintained 4 years post-rotational osteotomy. This was not possible pre-osteotomy

The gait pattern revealed an outward foot progression angle of at least 30° preoperatively. Patients were unable to sit upright in a chair and lean forward to tie their shoe laces. The preoperative examination included a standard torsional profile measured in the prone position. Inward rotation of the involved hip was limited to 0° (or even less) typically as compared to outward rotation >90°. Based on these findings, 45° of rotational correction was undertaken at the time of the midshaft osteotomy. In the supine position, the range of hip flexion (<25°—patella neutral) was compared to flexion past 90° when the hip was rotated 45° outward (Fig. 2). This small retrospective series of patients did not lend itself to patient-reported outcomes or functional hip or knee scores.

### Imaging

Plain radiographs included a full-length standing AP legs (to assess the mechanical axis and relative limb lengths) along with a standing AP of the pelvis and frog lateral view. Each hip was judged to be a stable SCFE of intermediate severity. We did not grade the slip angle specifically because of the likelihood of projectional artifact and inter-observer error. Advanced imaging, such as MRI or CT scan with 3D reconstruction, was not undertaken as the osteotomy was away from the hip joint and should not change angles or coverage. We reasoned the abnormal head–neck offset could be dealt with subsequently, as needed, via hip arthroscopy. This has been undertaken in two of the hips and a third is pending.

### Gait analysis

**Fig. 3:**
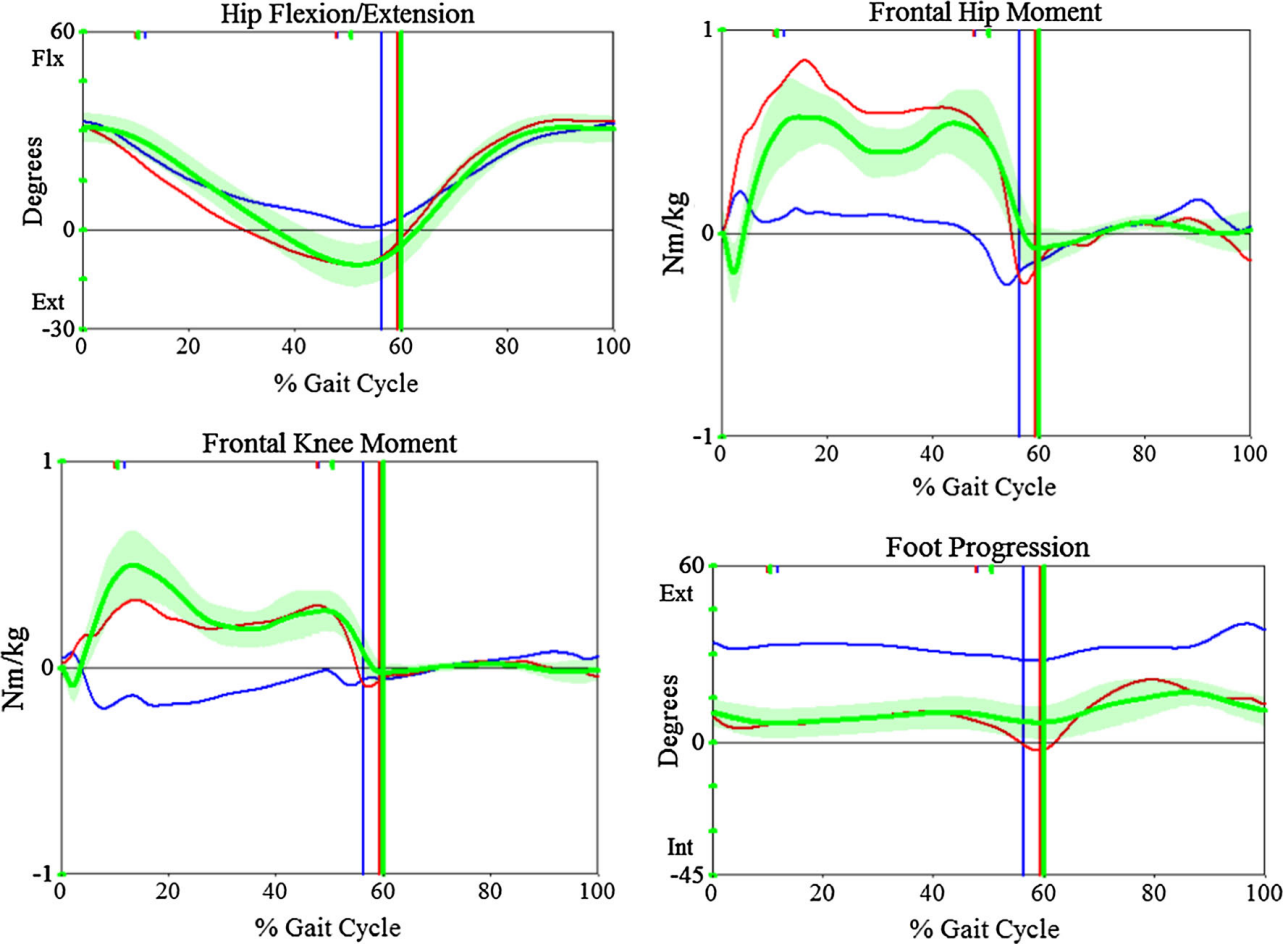
Movement analysis graphs depicting improvement, not only at the hip, but the ipsilateral knee as shown −/*blue* + preoperative/*red* = post-anteverting osteotomy/*green* = control (color figure online)

Pre- and post-operative computational gait analysis was performed on three of four subjects. A standard marker model was applied, and data were collected using a ten camera motion capture system (Vicon, Centennial CO, USA) and four force platforms (AMTI, Watertown, MA, USA) as the subjects ambulated at a self-selected velocity along a 10-m walkway [4]. Temporal parameters, kinematics and kinetics of the hip, knee and ankle, and an overall measure of gait kinematics are reported using the gait deviation index (Fig. 3) [4].

### Surgical technique for FRSO

The patient was placed supine on a standard radiolucent table with a radiolucent bump under the ipsilateral hip. The degree of hip flexion, with the patella forward, was noted and compared to hip flexion with the knee rotated 45° of outward. In each case, flexion past 90° was obtained (Fig. 2a, b). A trochanteric entry intramedullary rod, avoiding the piriformis fossa penetration, was utilized for stabilization post-osteotomy. Rotational guide pins (7/64 Steinman pins) were inserted into the proximal and distal femur to anticipate inward rotation correction of 45°. The osteotomy was performed percutaneously, at the midshaft level, connecting transverse drill holes with an Ilizarov osteotome and applying torque with a wrench. The distal interlocking screw was inserted first, using the perfect circle method, followed by the proximal screw via a jig [5]. Patients were permitted progressive weight bearing with crutches and physical therapy was utilized when needed to address abductor and quadriceps weakness and range of motion.

## Results

The mean follow-up was 44 months (range 24–74 months). The prone rotational profile was improved uniformly with an average of 40° inward and 60° of outward rotation of the hips (as compared to 0° inward and 90° outward preoperatively). The foot progression angle had improved from 35° outward to within normal range (0°–15°). Hip flexion, limited to 20° preoperatively, was maintained past 90° with the knee(s) in neutral. The patients were able to sit in a chair, with the knee(s) facing forward and bend over to tie their shoe laces (Fig. 2d).

There were no intra-operative or immediate postoperative complications. Osteonecrosis was absent on follow-up. There were two long-term postoperative complications; both male patients experienced interlocking screw fatigue failure and some loss of rotational correction requiring revision intramedullary fixation at 5 and 8 months after the index procedure. In both cases, the desired rotational correction was achieved ultimately. The interlocking screw failure has since been addressed by a redesign of the core diameter of 3.0–3.75 mm.

One patient underwent an arthroscopic osteochondroplasty in conjunction with extraction of screws and rod. Another patient underwent the same for ongoing impingement symptoms 6 months after rotational osteotomy. This patient had hip chondrolysis on presentation. The first (and oldest) patient eventually underwent a total hip arthroplasty at age 23, 5 years after rotational osteotomy. The joint surgeon reported no challenges or extenuating circumstances resulting from the prior diaphyseal osteotomy.

### Gait study

This series was too small to establish significant differences from pre- and postoperative gait analysis (Fig. 3). The investigation remains informative on the overall impact of retroversion from chronic SCFE. Knee pain that is commonly associated with SCFE is probably due to the retroversion which has a negative impact upon knee kinematics. As a result, pre- and postoperative torsional profiles and comparative gait analysis are planned for a group of patients undergoing arthroscopic osteochondroplasty.

## Discussion

The proximal femoral abnormalities characteristic of SCFE are a well-known cause of femoroacetabular impingement (FAI) [6–8]. The pistol grip deformity from hip varus and retroversion causes a decreased head/neck offset [9]. This deformity causes abutment of the head–neck junction within the acetabulum which leads to intra-articular chondrolabral damage frequently [10–12]. Typically, the degree of slip is graded according to plain AP and frog lateral radiographic images, but these fail to illustrate the acquired retroversion from SCFE. While CT scans with 3D reconstruction may demonstrate the complex nature of this deformity, these imaging modalities are typically focused upon the hip and do not include the distal femur. Consequently, the contribution of retroversion is not recognized. It is the clinical examination that puts the acquired retroversion and knee malorientation in perspective; this should include noting the foot progression angle, the prone torsional profile and the range of hip flexion with the knee neutral and limb rotated outward. This correlates well with the forced outward orientation of the limb when attempting to flex the knee and hip in the supine position. It explains the obligate out-toeing and altered knee kinematics and pain that should be addressed in corrective surgery.

The treatment approach to the patient with symptomatic FAI secondary to malunited SCFE has focused on the proximal femur and remains controversial. Treatment strategies range from watchful waiting to surgical intervention which has included one or more of the following: arthroscopy, intertrochanteric osteotomy, surgical dislocation and osteochondroplasty, arthrodesis and total hip arthroplasty [13, 14]. Fabricant et al. [15] noted that treatment of FAI may have less benefit in patients with relative femoral retroversion. Several groups have demonstrated clinical improvement with intertrochanteric osteotomy and base of neck extra-capsular osteotomies [16, 17]. Surgical dislocation (or arthroscopy) with osteochondroplasty alone can be used to improve the head–neck offset and address chondrolabral lesions. However, the reported outcomes have focused on pain relief and improvement in femoral head-to-neck offset. Leunig et al. [18] advocated for simultaneous in situ pinning and osteochondroplasty of the head–neck junction to improve offset in mild SCFE deformities. However, this technique lacks the power to resolve the obligate out-toeing that is so often associated with severe slips and may not improve knee kinematics.

Subcapital correction osteotomy has gained attention recently because it permits correction of both the varus and retroversion deformity of the proximal femur without creating other deformities. Numerous studies have discussed the use of subcapital cuneiform osteotomies to correct SCFE deformities in skeletally immature patients with concerning rates of AVN and chondrolysis [19–22]. Ziebarth and Huber each reported on a series of modified Dunn osteotomies and Slongo reported on open reductions in acute SCFE’s, each utilizing the surgical dislocation technique described by Ganz with minimal risk of AVN and chondrolysis [10, 23]. However, a recent series on the subcapital correction osteotomy for SCFE deformities in patients with closed physes utilizing the surgical dislocation technique reported a concerning rate of AVN despite good results in the majority of patients [24].

Intertrochanteric osteotomy has been used to address varus and retroversion deformities; however, it has its own set of complications including AVN, nonunion and malunion [25]. For the surgeon who uses this as their “go to” method to address severe SCFE deformities, we would suggest on making a goal of gaining 35°–45° of anteversion through rotation of the fragments in addition to the valgus correction gained. We believe the retroversion is the greater cause of impingement and obligate out-toeing when walking and sitting while the varus deformity is more related to the limb length inequality and trochanteric impingement.

While femoral shaft rotational osteotomy does not address the proximal femoral deformity directly, this technique recovers a functional range of hip motion and allows them to walk with their “hip” externally rotated (but foot neutral), thereby mitigating the effects of FAI. This also allows patients to sit more naturally as demonstrated by the improvement in hip flexion and normalization of their internal–external rotational range from their hips, both prone and at 90° of flexion. This osteotomy is comparatively safe and well tolerated with no associated risks of iatrogenic necrosis or chondrolysis.

### Limitations

Our study has several limitations. This is a retrospective series of a small number of patients with short follow-up. Given the lack of a control group and short follow-up, we cannot comment on the influence on hip arthritis. Furthermore, we recognize that a rotational osteotomy alone does not address the head–neck offset abnormalities or chondrolabral pathology of the acetabulum. However, if the patient continues to manifest symptoms (three of five hips in this series), these issues can be addressed either at the time of derotation or subsequently, through a small Smith–Peterson approach or arthroscopically if the patient continues to be symptomatic. Finally, we had two nonunions/delayed unions, with loss of correction, treated with revised reamed nailing. These complications are related to choice of hardware rather than a flawed surgical strategy. Both patients broke single 4.5 mm screws (core diameter of 3.0 mm) and appeared to have lost enough of the rotational correction to warrant revision surgery. While we have had success using single interlock screws both proximal and distal for rotational osteotomies in miserable malalignment patients, we have found that SCFE patients (more often male and larger) are at an increased risk of fixation failure [26]. We now use a 3.75-mm-core-diameter bolt preferentially.

Due to the acquired retroversion, patients with symptomatic FAI secondary to SCFE deformities have significant functional limitations, including obligate outward rotation when walking and during hip flexion. Femoral shaft rotational osteotomy is a familiar and safe technique that may normalize knee kinematics, while improving gait and hip flexion, hip biomechanics and short-term hip outcomes, without posing the risk of serious complications.
